# Pulmonary *Pseudomonas aeruginosa* infection induces autophagy and proteasome proteolytic pathways in skeletal muscles: effects of a pressurized whey protein-based diet in mice

**DOI:** 10.1080/16546628.2017.1325309

**Published:** 2017-06-01

**Authors:** Osama A. Kishta, Yeting Guo, Mahroo Mofarrahi, Flavia Stana, Larry C. Lands, Sabah N. A. Hussain

**Affiliations:** ^a^ Respiratory Division, Department of Medicine, Montréal Children’s Hospital, McGill University Health Centre, Montréal, QC, Canada; ^b^ Department of Pediatrics, McGill University, Montréal, QC, Canada; ^c^ Department of Critical Care, McGill University Health Centre, Montréal, QC, Canada; ^d^ Meakins-Christie Laboratories, Department of Medicine, McGill University, Montréal, QC, Canada

**Keywords:** Skeletal muscles, catabolism, autophagy, proteasome, whey protein, cystic fibrosis, *Pseudomonas aeruginosa* infection

## Abstract

**Background**: Pulmonary *Pseudomonas aeruginosa* infection in cystic fibrosis patients is associated with skeletal muscle atrophy. In this study, we investigated the effects of *P. aeurginosa* infection and a whey protein-rich diet on skeletal muscle proteolytic pathways.

**Design**: An agar bead model of pulmonary *P. aeurginosa* infection was established in adult C57/Bl6 mice. Protein ubiquitinaiton, lipidation of LC3B protein and expressions of autophagy-related genes and ubiquitin E3 ligases were quantified using immunoblotting and qPCR. The effects of pressure-treated whey protein diet on muscle proteolysis were also evaluated.

**Results**: Pulmonary *P. aeurginosa* infection reduced diaphragm, tibialis anterior, and soleus muscle weights and increased protein ubiquitination, LC3B protein lipidation, and the expressions of *Lc3b*, *Gabarapl1*, *Bnip3*, *Parkin, Atrogin-1*, and *MuRF1* genes in each muscle. These changes were greater in the tibialis as compared to soleus and diaphragm. Proteolysis indicators increased within one day of infection but were not evident after seven days of infection. A pressurized whey diet attenuated LC3B protein lipidation, expressions of autophagy-related genes (BNIP3), pro-inflammatory cytokines, and protein ubiquitination.

**Conclusions**: We conclude that pulmonary *P. aeruginosa* infection activates the autophagy, and the proteasome pathways in skeletal muscles and that a pressurized whey protein diet attenuates muscle proteolysis in this model.

## Introduction

Patients with cystic fibrosis (CF) suffer from weight loss, peripheral muscle atrophy, and impaired respiratory function [1–3]. They often contract chronic *Pseudomonas aeruginosa* lung infections. The same effects on weight, atrophy, and respiration are seen in mice with sustained *P. aeruginosa* infections [4–6]. In the diaphragm, infection is associated with increases in inflammatory cytokine expression [tumor necrosis factor-α (TNF-α), interleukin-1β (IL-1β), IL-6, and IL-18] and decreases in contractile performance [7].

Skeletal muscle protein degradation occurs as a consequence of the combined action of four pathways: the calpain, caspase, ubiquitin–proteasome, and autophagy–lysosome pathways. Calpains and caspases are involved in muscle protein degradation and operate under catabolic conditions by breaking down sarcomeric proteins [8]. The ubiquitin–proteasome pathway is mostly responsible for myofilament protein degradation, particularly in response to steroid therapy, denervation, and starvation, and is activated in the diaphragms of pulmonary *P. aeruginosa*-infected mice [9]. The autophagy–lysosome pathway is a highly conserved proteolytic process in which proteins and organelles, such as the mitochondria, peroxisomes, and lipid vacuoles, are sequestered in double-membrane vesicles called autophagosomes and delivered to lysosomes for degradation and subsequent recycling [10]. Autophagy is a critical regulator of protein homeostasis and mitochondrial quality control in normal skeletal muscles and is significantly induced in response to denervation, fasting, and severe sepsis [10,11].

Although it has generally been regarded as a protective and adaptive pathway, under certain circumstances, such as severe oxidative stress, increased autophagy can lead to excessive proteolysis and atrophy and, as a result, contribute to significant muscle contractile dysfunction [12]. As yet, no information is available that demonstrates a clear association between pulmonary *P. aeruginosa* infection and enhanced autophagy in skeletal muscles. In response to other forms of stress, however, a study that we published in 2013 indicates that the degree of skeletal muscle autophagy that is induced in response to fasting varies between muscles, depending on their fiber-type distribution and oxidative capacity [13]. For example, autophagy was induced to a lesser extent in the diaphragm, which is rich in type I fibers, than in the tibialis anterior, which is rich in type II fibers [13].

The first objective of the present study was to investigate the effects of sustained pulmonary *P. aeruginosa* infection on autophagic and proteasomal proteolytic pathways in murine skeletal muscles. We hypothesized that significant inductions of these pathways would occur in skeletal muscles in response to *P. aeruginosa* and that the degrees of induction would be more severe in limb muscles than in the diaphragm. Autophagy was detected by measuring levels of the lipidated form of LC3B protein (LC3B-II), which is conjugated to phosphatidylethanolamine and incorporated into the autophagosomes. We also quantified messenger RNA (mRNA) and protein levels of various autophagy-related proteins, including BECN-1, BNIP3, and SQSTM1 (p62). The proteasome was quantified by measuring protein ubiquitination and the expression of muscle-specific ubiquitin E3 ligases (*Atrogin-1* and *MuRF1*).

Whey protein, a by-product of cheese production, is rich in essential amino acids, has strong antioxidant potential, and is able to enhance muscle accretion [14–17]. A modified product of whey protein has been developed by exposing whey to hyperbaric pressure, thereby improving its digestibility and antioxidant potential [18,19]. Application of high hydrostatic pressure enhances digestibility by rendering whey more susceptible to the effects of proteolytic enzymes, resulting in an altered peptide profile, the generation of low molecular weight peptides, and increased cellular glutathione levels, which, in turn, results in reduced inflammatory responses [19].

The second objective of this study was to compare the effects of native and pressurized whey protein diets on the induction of the autophagic and proteasomal proteolytic pathways in skeletal muscles of mice with pulmonary *P. aeruginosa* infection. We have previously shown that a pressurized whey-based diet fed to mice with sustained *P. aeruginosa* infection decreases lung bacterial burden and airway protein oxidation [5]. Since evidence from our laboratories indicates that reactive oxygen species (ROS) play an important role in inducing autophagy in skeletal muscles, in the present study we hypothesized that pressurized whey protein, which has stronger antioxidant properties than the native form, would attenuate autophagy and the proteasome [20].

## Methods

### Materials

Antibodies for BECN1 (Beclin-1), phosphotidylinositol-3-phosphate class III (PI3KC3), microtubule-associated protein 1 light chain 3 beta (LC3B), total and phosphorylated (Thr^172^) adenosine monophosphate-activated protein kinase-α (AMPKα), and total and phosphorylated p65 subunit of nuclear factor-κB (NFκB) p65 (Ser^536^) were obtained from New England Biolabs (Pickering, ON, Canada). Antibody for sequestosome-1 (p62/SQSTM1) was obtained from Abnova (Walnut, CA, USA). Antibodies for BLC2/adenovirus E1B 19 kDa interacting protein 3 (BNIP3) and β-TUBULIN were obtained from Sigma-Aldrich (Oakville, ON, Canada). Monoclonal anti-ubiquitin antibody was obtained from Covance (Princeton, NJ, USA).

### Animal experiments

All procedures were approved by the University Animal Care Committee (UACC) of McGill University and were in compliance with all Canadian Council on Animal Care ethical guidelines. Four- to six-week-old female C57BL/6 mice (Charles River Laboratories, St. Constant, QC, Canada) were housed at the McIntyre animal facility at McGill University in individually ventilated clean cages on 12 h light/dark cycles. To acclimatize to their new environment before being assigned to experimental groups, for 4–6 days they had access to fresh water and a standard commercial chow diet *ad libitum*. They were then assigned to a time-course study or a dietary intervention study (see Experimental protocols, below). Female C57BL/6 mice were used because they are more susceptible to pulmonary inflammation and infection with *P. aeruginosa* than other strains [21].

### Infection

*Pseudomonas aeruginosa* (mucoid strain 508) was embedded in agar beads as previously described [5]. The bacterial sample was originally extracted from the sputum of a CF patient and was a kind gift from Drs D. Radzioch and B. Petrof (McGill University). Mice were infected with 8 x 10^5^ cfu/mouse using a minimally invasive, direct visualization method that instilled *P. aeruginosa*-embedded agar beads directly into the deeper airways via the trachea, as previously described [4]. Sterile agar beads were instilled into the tracheas of control group animals.

### Diets and feeding protocol

Following an acclimatization period, mice were weighed and assigned semi-purified custom-made diets consisting of native (unpressurized) or pressurized whey protein isolate as the sole protein source (Inpro 90; Vitalus Nutrition, Abbotsford, BC, Canada). Whey composition on a dry weight basis consisted of greater than 92% protein. Smaller protein components included β-lactoglobulin (43–48%), glycomacropeptides (24–28%), α-lactalbumin (14–18%), bovine serum albumin (1–2%), immunoglobulins (1–3%), and lactoferrin (< 1%). Whey protein was pressure-treated as previously described [5]. It was then lyophilized at −40°C (Agriculture and Agri-Food Canada, St-Hyacinth, QC, Canada). Whey powder (native or pressurized) was incorporated at 20% (w/w) into semi-purified AIN-93 isocaloric diets obtained from MP Biomedical (Santa Ana, CA, USA). The composition of the diet is listed in Table 1. Diets were stored at −20°C until needed. Food consumption was monitored daily by measuring the weight of leftover food and replacing it with fresh food. Mice were fed for 4 weeks before they underwent pulmonary infection. During this feeding period, body weights of mice were recorded every other day to ensure normal growth. Mice gained 18–20% of their body weight from baseline (pre-feeding) with no observed differences between groups. Post-infection food consumption was monitored and recorded daily.

**Table 1.** Composition of the diet used in the current study.

Ingredient	w/w%
Corn starch	36.9
Pressurized or native whey protein	20
Dextrinized corn starch	10
Sucrose	9
Butter	2.8
Lard	2.8
Corn oil	1.4
Soybean oil	7
Alphacel non-nutritive bulk	5
AIN-93G mineral mix	3.5
AIN-93G vitamin mix	1
L-cystine	0.3
Choline bitartarate	0.25

### Experimental protocols

Three experiments were performed.

#### Evidence of autophagy

This experiment was designed to compare the degree of autophagy and pro-inflammatory cytokine production in three skeletal muscles: the tibialis anterior (TA), which is rich in glycolytic fibers; the diaphragm (DIA), which is representative of the ventilatory muscles; and the soleus (SOL), which is rich in oxidative fibers. Animals were examined on day 3 post-infection, when body weight loss, lung inflammation, and clinical features characteristic of this model peak [4,5].

#### Time course of autophagy and protein ubiquitination

This experiment was designed to assess the time course of autophagy and protein ubiquitination in the DIA in response to pulmonary *P. aeruginosa* infection. The DIA was chosen because it is the principal respiratory muscle and is known to be significantly affected by *P. aeruginosa* in the murine model [22]. Control (sterile beads) and *P. aeruginosa*-infected animals were killed on days 1, 3, and 7 post-infection and the DIA was quickly excised and stored in liquid nitrogen until further use.

#### Dietary intervention

This experiment was designed to compare the effects of two types of whey protein diet on autophagy and protein ubiquitination in skeletal muscles in response to pulmonary *P. aeruginosa* infection. Following an acclimatization period, mice were randomized to receive a semi-purified diet containing either native or pressurized whey protein for 4 weeks. Mice in each group were then infected intratracheally with 8 × 10^5^ cfu of *P. aeruginosa* and examined at days 1 and 3 post-infection.

### Protein extraction and immunoblotting

Frozen muscle samples were homogenized in buffer [10 mM Tris–maleate, 3 mM ethylene glycol-bis(β-aminoethyl ether)-*N,N,N*',*N*'-tetraacetic acid (EGTA), 275 mM sucrose, 0.1 mM dithiothreitol (DTT), 2 μg/ml leupeptin, 100 μg/ml phenylmethane sulfonyl fluoride (PMSF), 2 μg/ml aprotinin, and 1 μg/100 ml pepstatin A, pH 7.2]. Samples were centrifuged at 1000 × *g* for 10 min. Pellets were discarded and supernatants were designated as crude homogenates. Total muscle protein levels in each sample were determined using the Bradford protein assay technique. Crude homogenate samples (20 μg/sample) were mixed with sodium dodecyl sulfate (SDS) sample buffer, boiled for 5 min at 95°C, then loaded on to Tris–glycine SDS polyacrylamide gels. Proteins were transferred by electrophoresis to polyvinylidene difluoride (PVDF) membranes and blocked with 1% bovine serum albumin or milk for 1 h at room temperature. PVDF membranes were incubated overnight with primary antibodies at 4°C, then washed and incubated with horseradish peroxidase-conjugated secondary antibody. Specific proteins were detected with an enhanced chemiluminescence kit. Equal loading of proteins was confirmed by stripping membranes and re-probing with anti-β-TUBULIN antibody. Blots were scanned with an imaging densitometer and optical densities (ODs) of protein bands were quantified using Gel-Pro® Analyzer software (MediaCybernetics, Rockville, MD, USA).

### RNA extraction and real-time polymerase chain reaction (PCR)

Total RNA was extracted from muscle samples using a GenElute™ Mammalian Total RNA Miniprep Kit (Sigma-Aldrich, Oakville, ON, Canada). Quantification and purity of total RNA was assessed by *A*_260_/*A*_280_ absorption. Total RNA (2 µg) was reverse transcribed using a Superscript II® Reverse Transcriptase Kit and random primers (Invitrogen Canada, Burlington, ON, Canada). Reactions were incubated at 42°C for 50 min and at 90°C for 5 min. Real-time PCR detection of mRNA expression was performed using a Prism® 7000 Sequence Detection System (Applied Biosystems, Foster City, CA, USA). mRNA expression of autophagy-related genes was assessed owing to their importance to: (i) the initial phase of autophagosome formation (*Pi3kc3*); (ii) the expansion of the isolation membrane (*Lc3b*, *Gabarapl1*); (iii) the induction of mitophagy (*Parkin*); (iv) chaperon-mediated autophagy by lysosomal-associated membrane protein 2 (*Lamp2a*); (v) Forkhead box O1 (*Foxo1*) regulation of autophagy; (vi) targets of *Foxo1* transcription factor (*Bnip3)*; and (vii) the muscle-specific E3 ligases F-box protein 32 (*Atrogin-1*) and Muscle RING finger 1 (*MuRF1*). Specific sets of primers used to detect genes are listed in Table 2. In all assays, 1.0 µl of reverse-transcriptase reagent was added to 25 µl of SYBR® Green master mix (Qiagen, Valencia, CA, USA) and 3.5 µl of 10 µM primer. The thermal profile used was: 95°C for 10 min; 40 cycles each of 95°C for 15 s; 57°C for 30 s; and 72°C for 33 s. All real-time PCR experiments were performed in triplicate. A melt analysis for each PCR experiment was performed to assess primer–dimer formation or contamination. Quantification of relative mRNA levels of target genes was determined using the threshold cycle (ΔΔ^CT^) method using the housekeeping gene *β-Actin*, as described in our previous study [11].

**Table 2.** Primers used in real-time polymerase chain reaction experiments to detect expression of various autophagy-related genes in the tibialis anterior, diaphragm, and soleus muscles.

Gene			Accession no.
*Atrogin-1*	Forward	5’-TGGGTGTATCGGATGGAGAC-3’	NM_026346
	Reverse	5’-TCAGCCTCTGCATGATGTTC-3’	
*β-Actin*	Forward	5’-CTGGCTCCTAGCACCATGAAGAT-3’	NM_007393
	Reverse	5’-GGTGGACAGTGAGGCCAGGAT-3’	
*Bnip3*	Forward	5’-TTCCACTAGCACCTTCTGATGA-3’	NM_009760
	Reverse	5’-GAACACCGCATTTACAGAACAA-3’	
*Foxo1*	Forward	5’-AAGGATAAGGGCGACAGCAA−3’	NM_19739
	Reverse	5’-TGGATTGAGCATCCACCAAG−3’	
*Gabarapl1*	Forward	5’-CATCGTGAGAAGGCTCCTA-3’	NM_020590
	Reverse	5’-ATACAGCTGGCCCATGGTAG-3’	
*IL-6*	Forward	5’-CACGGCCTTCCCTACTTCAC-3’	NM_020590
	Reverse	5’-TGCAAGTGCATCATCGTTGT-3’	
*Lamp2a*	Forward	5’-TGGCTAATGGCTCAGCTTTC-3’	NM_031168
	Reverse	5’-ATGGGCACAAGGAGTTGTC-3’	
*Lc3b*	Forward	5’-CGATACAAGGGGGAGAAGCA-3’	NM_026160
	Reverse	5’-ACTTCGGAGATGGGAGTGGA-3’	
*MuRF1*	Forward	5’-AGAAGCTGGGCTTCATCGAG−3’	NM_1039048
	Reverse	5’-TGCTTGGCACTTGAGAGGAA−3’	
*Parkin (Park2)*	Forward	5’-AACTGTGACCTGGAACAACA-3’	NM_01664
	Reverse	5’-CTGGACCTCTGGCTGCTTCT-3’	
*Pik3c3*	Forward	5’-TGTCAGATGAGGAGGCTGTG-3’	NM_181447
	Reverse	5’-CCAGGCACGACGTAACTTCT-3’	
*Sqstm1* (p62)	Forward	5’-GCACCTGTCTGAGGGCTTCT-3’	NM_003900
	Reverse	5’-GCTCCAGTTTCCTGGTGGAC-3’	
*TNF*α	Forward	5’-ACTGGCAGAAGAGGCACTCC-3’	NM_013693
	Reverse	5’-CTCCAGCTGCTCCTCCACTT-3’	

### Statistical analysis

Data are expressed as means ± SEM. Two-way analysis of variance (ANOVA) followed by a Holm–Ŝídák post-hoc test was used for all pairwise multiple comparisons. For feeding interventions, a paired *t* test was used to compare native and pressurized whey diets. Significance was set at *p* < 0.05, tendency was defined as greater than or equal to 0.1. SigmaPlot™ 11 was used to graph the data.

## Results

### Evidence of autophagy

**Figure 1.**
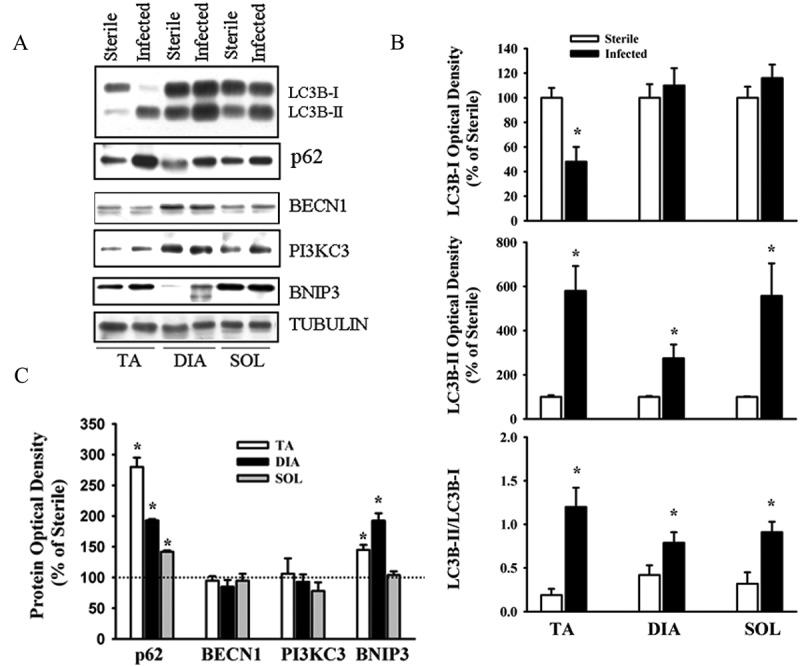
(A) Representative immunoblots of various autophagy-related proteins; (B, C) optical densities of (B) LC3B-I, LC3B-II, and LC3B-II/LC3B-I protein ratios, and (C) SQSTM1 (p62), BECN1, PI3KC3, and BNIP3, in murine tibialis anterior (TA), diaphragm (DIA), and soleus (SOL) muscles 3 days after pulmonary instillation of sterile (control) or *Pseudomonas aeruginosa*-infected agar beads. Values are means ± SEM, expressed as a percentage of control values. *p < 0.05 compared to control (sterile bead) group. *n* = 5 for TA and SOL, *n* = 7 for DIA.

**Figure 2.**
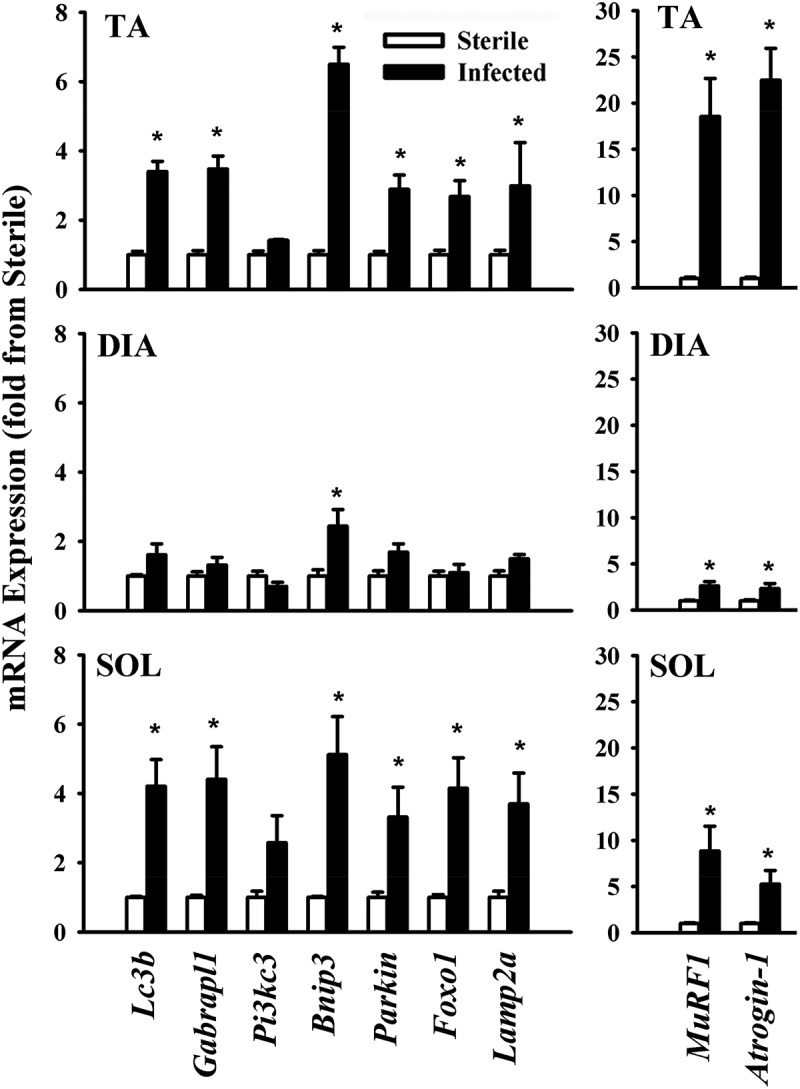
Messenger RNA (mRNA) expression of various autophagy-related genes and the muscle-specific E3 ligases *Atrogin-1* and *MuRF1* in murine tibialis anterior (TA), diaphragm (DIA), and soleus (SOL) muscles 3 days after pulmonary instillation of *Pseudomonas aeruginosa*-infected agar beads. Values are means ± SEM. *p < 0.05 compared to control (sterile bead) group. *n* = 6 per group.

**Figure 3.**
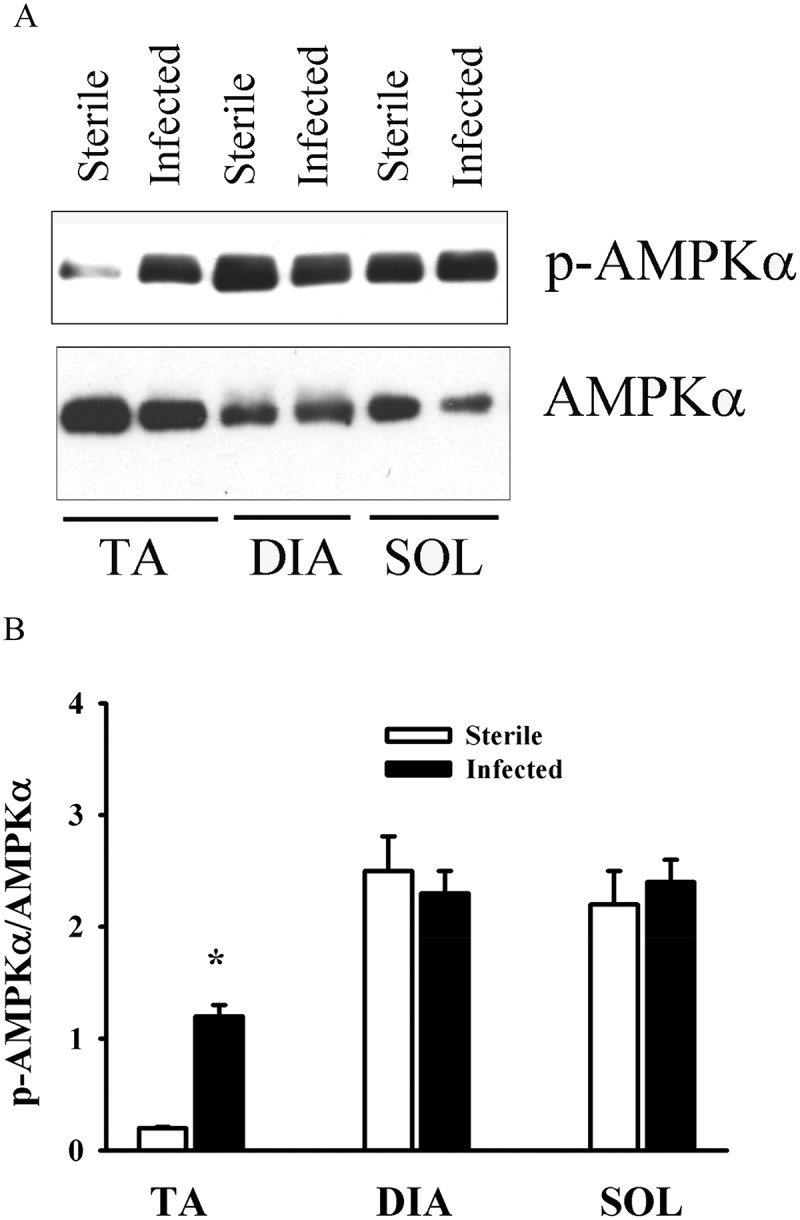
(A) Representative immunoblots, and (B) optical densities of phosphorylated (p-) and total AMP-activated protein kinase (AMPK) protein in murine tibialis anterior (TA), diaphragm (DIA), and soleus (SOL) muscles 3 days after pulmonary instillation of *Pseudomonas*. *aeruginosa*-infected agar beads. Values are means ± SEM, expressed as fold change relative to control values. *p < 0.05 compared to control (sterile bead) group. *n* = 5 per group.

**Figure 4.**
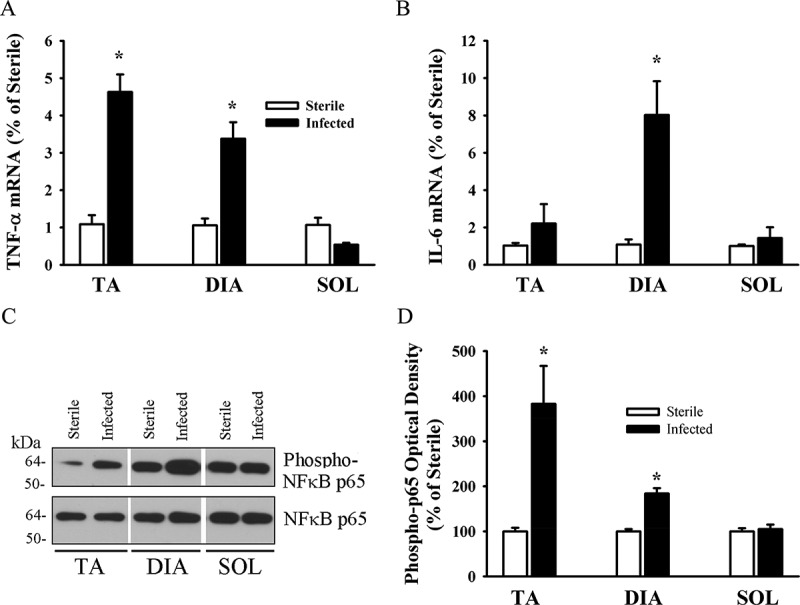
Messenger RNA (mRNA) expression of (A) tumor necrosis factor-α (*TNF-α*) and (B) interleukin-6 (*IL-6*) in murine tibialis anterior (TA), diaphragm (DIA), and soleus (SOL) muscles 3 days after pulmonary instillation of *Pseudomonas aeruginosa*-infected agar beads. Values are means ± SEM, expressed as fold change relative to control values. *p < 0.05 compared to control (sterile bead) group. *n* = 6 per group. (C) Representative immunoblots, and (D) optical densities of phosphorylated (Phospho-) and total nuclear factor-κB (NFκB) protein in murine TA, DIA, and SOL muscles 3 days after pulmonary instillation of *P. aeruginosa*-infected agar beads. Values are means ± SEM, expressed as percentage change relative to sterile values. *p < 0.05 compared to control (sterile bead) group. *n* = 4 per group.

Food intake in the *P. aeruginosa*-infected groups declined by days 1 and 3 post-infection, averaging 55.2 ± 15.5% and 41.5 ± 11.4% of the control group value, respectively. Changes in food intake were associated with relatively small, albeit significant (*p* < 0.05 compared to control), decreases in body weight (an average of 6% and 8% by days 1 and 3, respectively). At day 3, TA, DIA, and SOL wet weights averaged 74%, 62%, and 68% of the control value, respectively (*p* < 0.05 compared to control). At day 7, there were no differences in food intake, body weight, or individual muscle weight between the infected and control groups. At day 3, the intensity of lipidated LC3B protein (LC3B-II) increased and the intensity of the free form of LC3B (LC3B-I) decreased in the TA compared to the control group (Figure 1). Relative increases in LC3B lipidation and LC3B-II/LC3B-I ratios in response to infection were higher (> 5-fold) in the TA and SOL compared to the DIA (3-fold) (Figure 1). In all three muscles, infection induced increases in p62/SQSTM1 and BNIP3 protein levels, but exerted no influence on BECN1 or PI3KC3 (Figure 1). The relative increase in p62/SQSTM1 protein was significantly higher (2.5-fold) in the TA than in the DIA or SOL (Figure 1). At day 3 in the TA and SOL muscles, infection triggered increases in the mRNA expression of several autophagy-related genes, including *Lc3b*, *Gabarapl1, Bnip3*, *Parkin, Lamp2a*, and *Foxo1* (Figure 2). In the DIA, only *Bnip3* mRNA levels increased (Figure 2). In all three muscles, infection triggered increases in the mRNA expression of two muscle-specific E3 ligases, *MuRF1* and *Atrogin-1* (*p* < 0.01) (Figure 2). Relative increases in their expression were higher in the TA and SOL than in the DIA (Figure 2). Phosphorylation of AMPKα on Thr^172^ was induced in the TA of infected animals, indicating that the AMPK pathway was activated in response to pulmonary *P. aeruginosa* infection (*p* < 0.01) (Figure 3). At day 3, muscle-derived *TNF-α* mRNA levels increased in the TA and DIA but not in the SOL (*p* < 0.01) (Figure 4). Muscle-specific *IL-6* mRNA levels increased only in the DIA (Figure 4). At day 3, phosphorylation of p65 (RelA) on Ser^536^ was induced in the TA and DIA of infected animals, indicating that the transcription factor NFκB was activated in response to *P. aeruginosa* infection (Figure 4).

### Time course of autophagy

**Figure 5.**
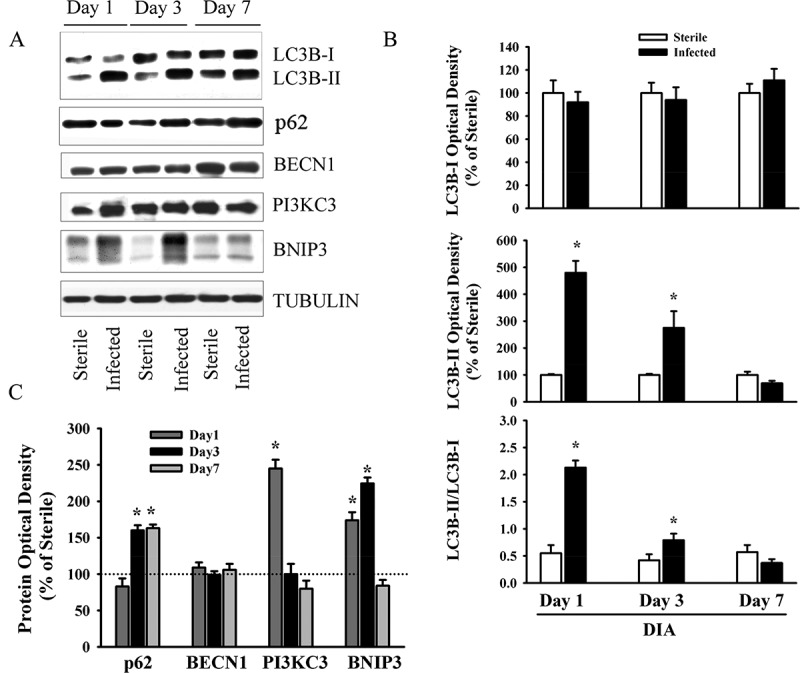
(A) Representative immunoblots, and (B, C) optical densities of (B) LC3B-I, LC3B-II, LC3B-II/LC3B-I ratios and (C) various autophagy-related proteins in murine diaphragm (DIA) muscle 1, 3, and 7 days after pulmonary instillation of *Pseudomonas aeruginosa*-infected agar beads. Values are means ± SEM, expressed as fold change relative to control values. *p < 0.05 compared to control (sterile bead) group. *n* = 6 per group.

**Figure 6.**
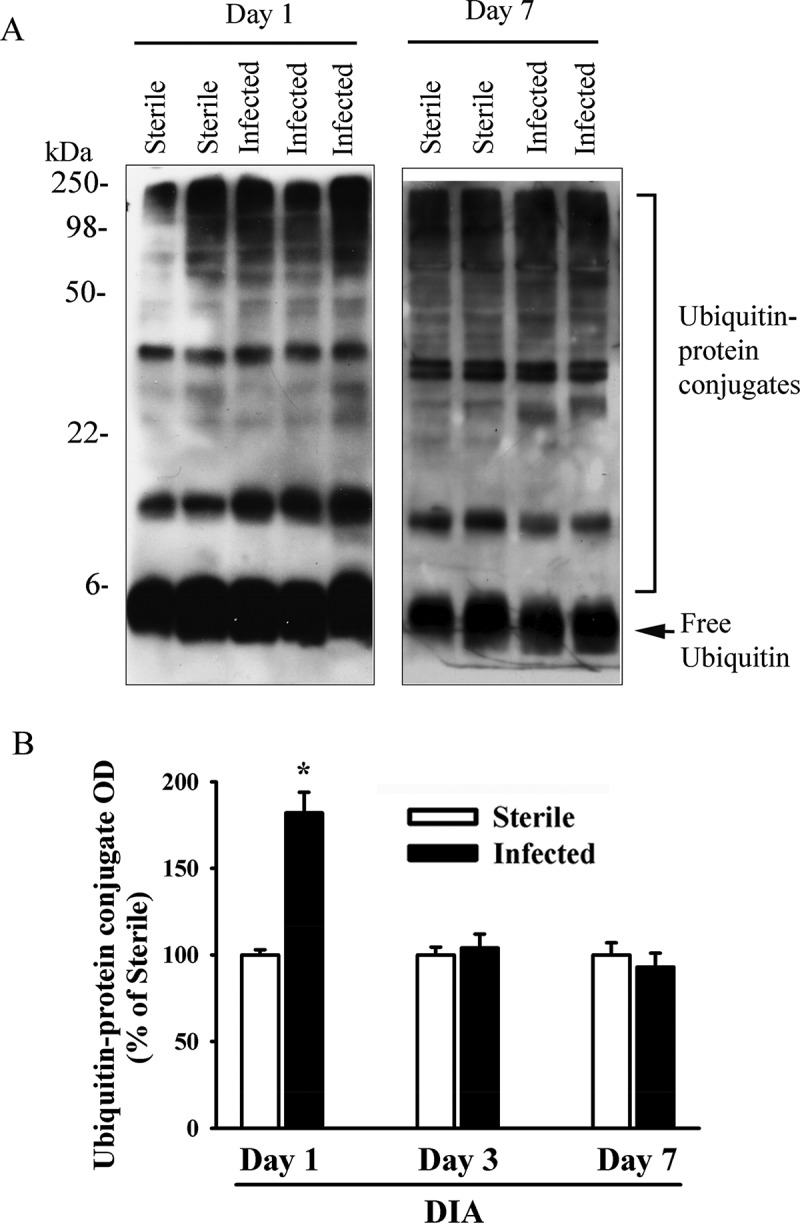
(A) Representative immunoblots, and (B) optical densities (OD) of ubiquitin–protein conjugate formation in murine diaphragm (DIA) muscle 1, 3, and 7 days after pulmonary instillation of *Pseudomonas aeruginosa*-infected agar beads. Values are means ± SEM, expressed as percentage of control values. *p < 0.05 compared to control (sterile bead) group. *n* = 6 per group.

Figure 5 illustrates changes in LC3B lipidation and several autophagy-related proteins in the DIA muscle during the course of pulmonary *P. aeruginosa* infection. At day 1, LC3B lipidation, as indicated by the intensity of the LC3B-II protein band, and LC3B-II/LC3B-I ratios increased (approximately 5- and 4-fold, respectively). LC3B protein lipidation increased to a lesser degree at day 3 and returned to control levels at day 7 (Figure 5). In addition to enhanced LC3B protein lipidation, increases in PI3KC3 and BNIP3 protein levels were observed in the DIA muscle at days 1 and 3. p62/SQSTM1 protein levels increased at days 3 and 7 (Figure 5). No changes in BECN1 levels were observed (Figure 5). To investigate a possible connection between increased autophagy in the DIA and increased proteasome activity, ubiquitin–protein conjugate levels were measured. Significant increases in these levels were observed (*p* < 0.05) (Figure 6), particularly at day 1 and, to a lesser extent, at day 3. These results indicate that pulmonary *P. aeruginosa* infection triggers increases in autophagy and activates proteolytic pathways.

### Dietary intervention

As would be expected with a model of sustained inflammation, the animals were anorexic. Daily food consumption at days 1 and 3 in the group fed native whey protein declined to 10.7 ± 2.9% and 30.6 ± 8.7%, respectively, of that seen in uninfected animals. Similarly, daily food consumption at days 1 and 3 in the group fed pressurized whey protein declined to 9.8 ± 2.1% and 24.3 ± 6.7% of that seen in uninfected animals. At no time were any differences in food intake observed between animals on the native and those on the pressurized whey protein diets.

**Figure 7.**
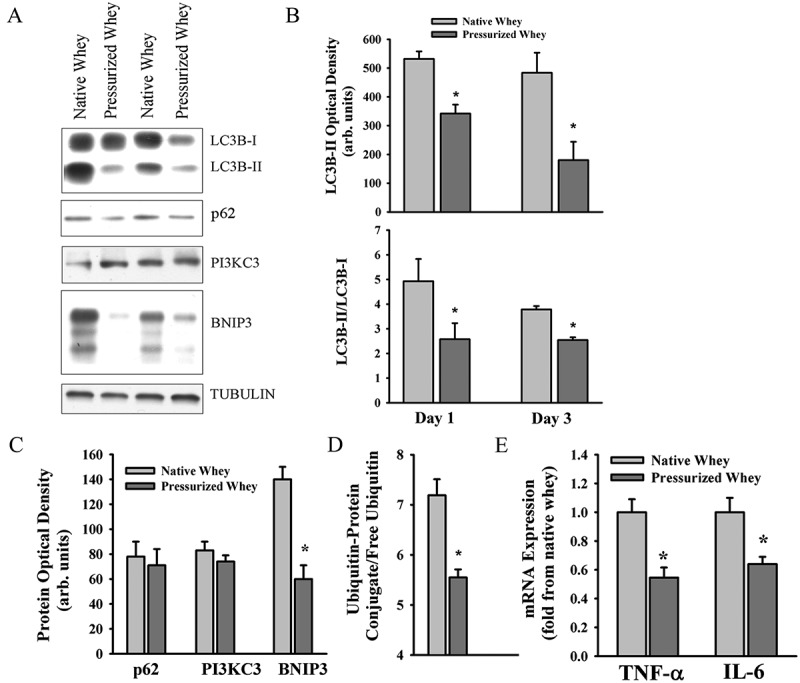
(A) Representative immunoblots, and (B–D) optical densities of (B) LC3B-II and LC3B-II/LC3B-I ratios, (C) autophagy-related proteins, and (D) ubiquitin–protein conjugate formation; (E) messenger RNA (mRNA) expression levels of tumor necrosis factor-α (*TNF-α*) and interleukin-6 (*IL-6*) in murine diaphragm (DIA) muscle 3 days after pulmonary instillation of *Pseudomonas aeruginosa*-infected agar beads in mice fed native (control) or pressurized whey protein diets. Values are means ± SEM. *p < 0.05 compared to control (native whey) diet. *n* = 6 per group.

**Figure 8.**
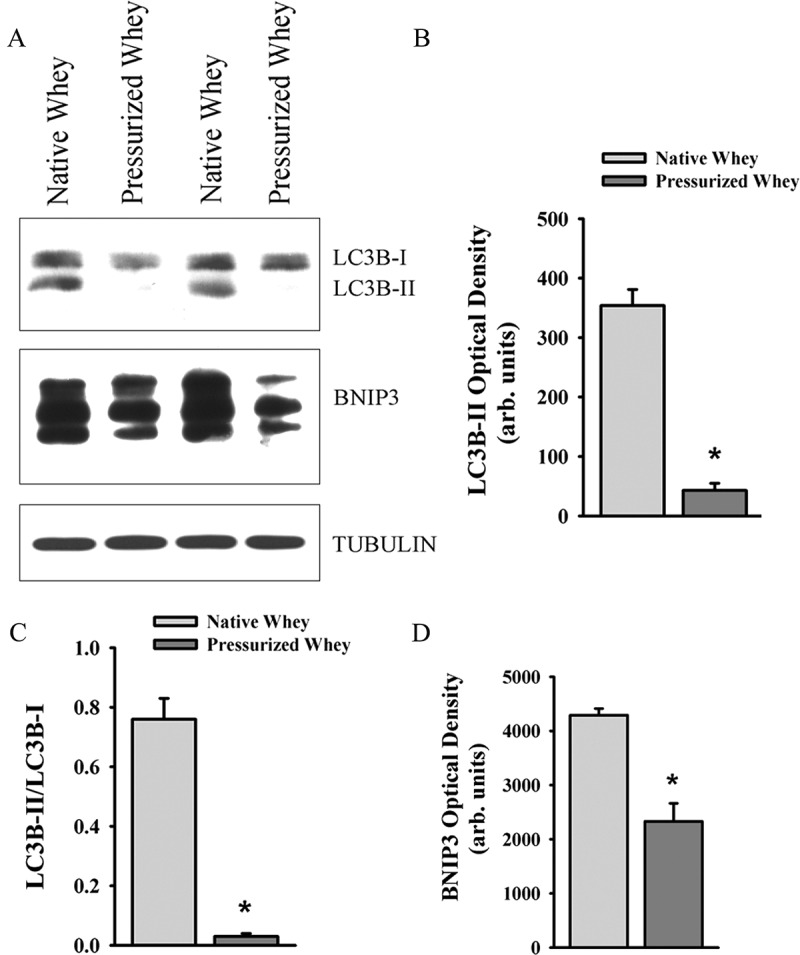
(A) Representative immunoblots, and (B–D) optical densities of (B) LC3B-II, (C) LC3B-II/LC3B-I ratios, and (D) BNIP3 proteins in murine tibialis anterior (TA) muscle 3 days after pulmonary instillation of *Pseudomonas aeruginosa*-infected agar beads in mice fed native (control) or pressurized whey protein diets. Values are means ± SEM. *p < 0.05 compared to control (native whey) diet. *n* = 5 per group.

LC3B protein lipidation levels in the DIA at days 1 and 3 were lower in the group fed pressurized whey compared to those on native whey (Figure 7). Levels of BNIP3 protein and ubiquitin–protein conjugate and levels of *TNF-α* and *IL-6* mRNA levels in the DIA were lower at day 3 in the group fed pressurized whey (Figure 7), as were levels of LC3B-II and BNIP3 in the TA (Figure 8). These results suggest that, in response to *P. aeruginosa* infection, a diet of pressurized whey attenuates activation of the autophagy–lysosome and ubiquitin–proteasome proteolytic pathways.

## Discussion

Pulmonary *P. aeruginosa* infection in mice is associated with significant body weight loss from days 1 to 3 post-infection [5,6,21]. We have shown that this weight loss does not persist at day 7 post-infection [5]. Weight loss in this model is also associated with significant reductions in skeletal muscle mass [6]. Muscle mass is regulated by the balance between protein synthesis and protein degradation, and the present study demonstrates that several autophagy- and protein ubiquitination-related proteins are significantly induced in the TA, DIA, and SOL muscles 1–3 days after pulmonary *P. aeruginosa* infection, indicating that proteolysis is induced and that this enhanced proteolysis may be a contributor to body weight loss.

We found that pulmonary *P. aeruginosa* infection was associated with increases in *Lc3b*, *Gabarapl1, Bnip3*, and *Parkin* mRNA expression, and enhanced p62/SQSTM1, BNIP3, and PI3KC3 protein levels (Figures 1, 2, and 5). These results are consistent with the notion that prolonged activation of autophagy in skeletal muscles triggers transcriptional up-regulation of the short-lived autophagy-related proteins that are required to sustain autophagosome formation [23]. Furthermore, induction of p62/SQSTM1, BNIP3, and *Parkin* in skeletal muscles in response to *P. aeruginosa* infection suggests that mitophagy was also elevated.

An important observation in our study is that *P. aeruginosa* elicits relatively strong inductions of LC3B lipidation, p62 protein expression, *Lc3b, Gabarapl1*, *Bnip3, Parkin*, and *Lamp2a* mRNA expression in the TA compared to the DIA (Figures 1 and 2). This finding is in accordance with our observations that there are significantly greater levels of basal, starvation-, and sepsis-induced autophagy in glycolytic muscles, such as the TA, compared to those rich in oxidative fibers, such as the DIA [11,13]. Mechanisms underlying these differences remain unclear. We speculate, however, that complex interactions involving transcriptional and post-transcriptional processes are likely to be involved [10]. At the transcriptional level, FOXO proteins have emerged as important factors that critically regulate specific autophagy-related genes such as LC3B, GABARAP, and BNIP3 when autophagy is sustained for relatively long periods [23,24]. In our study, we detected greater levels of *Foxo1* mRNA in the TA than in the DIA at day 3 post-infection, suggesting that *Foxo1* may contribute to the up-regulation of *Lc3b*, *Gabarapl1*, and *Bnip3* that is seen in the TA in response to infection (Figure 2). We should emphasize that other transcription factors, NFκB for example, may also contribute to the regulation of autophagy in skeletal muscles in infected animals. In fact, it has been shown in septic mice that selective inhibition of NFκB transcriptional activity results in significant attenuation of autophagy [11].

Studies indicate that sustained autophagy in skeletal muscles requires the activation of both a non-transcriptional program initiated by the ULK1/ATG13/FIP200 protein complex and a FOXO transcriptional program designed to up-regulate specific autophagy-related genes. At the post-transcriptional level, autophagy is regulated by several pathways, including the AMPK pathway, that facilitate the induction of autophagy through selective phosphorylation of several residues of ULK1, resulting in its dissociation from the inhibitory effects of mammalian target of rapamycin (mTOR) complex I and activation of its kinase activity, a critical step for autophagy initiation [25,26]. We investigated the role of AMPK activation in the induction of autophagy-related genes in skeletal muscles 3 days after infection with *P. aeruginosa*. We found that AMPKα phosphorylation on Thr^172^ (index of activation) was evident in the TA muscle only at day 3, suggesting that AMPK pathway activation in that muscle may be responsible, in part, for the induction of autophagy (Figure 3).

Another possible factor that may have contributed to the stronger induction of autophagy-related genes in the TA than in the DIA is the production of pro-inflammatory cytokines, particularly TNF-α and IL-6, which exert strong stimulatory effects on autophagy [27,28]. In this study, we found that at day 3 mRNA expression of muscle-derived *TNF-α* increased in the TA and DIA, but not in the SOL, while mRNA expression of muscle-derived *IL-6* increased only in the DIA by the same time point (Figure 4). These results concur with those of Divangahi et al. [7], who reported significant elevation of the pro-inflammatory cytokines *TNF-α*, *IL-6, IL-1α*, *IL-1β*, and *IL-18* in the DIA in response to pulmonary *P. aeruginosa* infection. Up-regulation of muscle-derived *TNF-α* and *IL-6* expression may therefore be an important factor regulating autophagy induction in response to *P. aeruginosa*, at least in the TA and DIA. It is important to note that pro-inflammatory cytokines other than TNF-α and IL-6, particularly IL-1β, may also be involved in the regulation of skeletal muscle autophagy in response to this type of pulmonary infection. Indeed, there is compelling evidence that IL-1β strongly activates autophagy [29].

An important observation in our study is that a pressurized whey diet strongly attenuates LC3B lipidation, BNIP3 protein expression, and ubiquitin–protein conjugate formation in the DIA and TA muscles, compared to a native whey diet. This suggests that the pressurization process alters the protein in a way that exerts a negative effect on the activities of the autophagy–lysosome and ubiquitin–proteasome proteolytic pathways (Figures 7 and 8). The mechanisms responsible for this effect remain unclear, but we speculate that three may be involved. First, it is possible that pressurized whey protein consumption may have attenuated the development of oxidative stress in these muscles. Proteins are major targets of ROS and their oxidation can alter functionality, including loss of enzymic activity and enhanced degradation. Increased levels of ROS have long been identified to be an important stimulus for increased proteolysis in skeletal muscles, and a previous study by this group confirmed that mitochondrial-derived ROS play an important stimulatory effect on autophagy in skeletal muscles [20]. Pressurized whey is known to strongly attenuate intracellular ROS formation in intestinal epithelial cells [18] and to reduce airway protein oxidation in mice infected with *P. aeruginosa* [5]. By extension, it may have exerted a similar effect in the present study.

Secondly, it is possible that the pressurized whey diet may have dampened systemic and local (muscle-derived) inflammatory responses. Previous reports have confirmed that this kind of diet reduces lung bacterial burden and levels of the airway inflammatory cytokines MIP-2 and KC (mouse homologues of IL-8) [5]. Supporting evidence for this theory includes the fact that IL-8 levels in CF patients tend to decrease when diets are supplemented with pressurized whey [30].

Thirdly, it is possible that improved digestibility of the protein brought about by the pressurization process led to increased availability of amino acids for gastrointestinal absorption. This theory is supported by the work of Vilela et al. [19], who reported a significantly higher amino acid profile in pressurized whey protein digestate compared to that from native whey protein. Increased amino acid absorption and enhanced serum amino acid levels would activate mTOR complex I, which, in turn, would significantly inhibit autophagy. This inhibition would occur by way of several mechanisms, including inhibition of ULK1 kinase activity, which, in turn, prevents autophagy initiation [25,26]. This interpretation is consonant with the improved nutritional status of CF patients whose diets have been supplemented with pressurized whey [30].

In summary, it is well established that exercise performance is significantly reduced in CF patients compared to healthy subjects and that their poor prognosis is associated with reduced exercise capacity [31,32]. Poor physical performance has been attributed, in part, to significant reductions in limb muscle strength [33,34]. Why poor limb muscle strength exists in CF patients is not entirely understood, although reduced muscle mass appears to be a major factor [34]. One major conclusion of the present study is that our results imply that the enhanced autophagy and proteasomal proteolysis that is seen in respiratory and limb muscles in response to infection plays an important role in reducing muscle mass and, therefore, contributes to the poor physical performance levels of CF patients.

Another major finding of our study is that a pressurized whey protein diet attenuates autophagy and ubiquitin–proteasome pathway induction in the diaphragm in response to *P. aeruginosa* infection. These results imply that diets that contain nutritionally derived products with antioxidant and anti-inflammatory properties that impinge upon skeletal muscle proteolysis, such as pressured whey protein, may have promising therapeutic potential in CF patients with acute pulmonary *P. aeruginosa* infection.
